# Writing direction and language activation affect how Arabic-English bilingual speakers map time onto space

**DOI:** 10.3389/fpsyg.2023.1356039

**Published:** 2024-01-24

**Authors:** Juana Park, Christina L. Gagné, Thomas L. Spalding

**Affiliations:** ^1^Department of Psychology, American University of Sharjah, Sharjah, United Arab Emirates; ^2^Department of Psychology, University of Alberta, Edmonton, AB, Canada

**Keywords:** metaphor, time, space, Arabic, bilingual, writing direction

## Abstract

We investigated whether writing direction and language activation influence how bilingual speakers map time onto space. More specifically, we investigated how Arabic-English bilingual speakers conceived where (e.g., on the left or on the right) different time periods (e.g., past, present, future) were located, depending on whether they were tested in Arabic (a language that is written from right to left) or in English (a language that is written from left to right). To analyze this, participants were given a task that involved arranging cards depicting different scenes of a story in chronological order. Results show that, when tested in Arabic, participants were significantly more likely to use right-to-left arrangements (following the Arabic writing direction), compared to when tested in English.

## Introduction

In this study, we explore whether writing direction and language activation influence how people map time onto space. To address this theoretical issue, we examined how Arabic-English bilingual speakers conceive where (e.g., on the left or the right) different time periods (e.g., past, present, future) are located, depending on whether they are tested in Arabic (a language that is written from right to left) or in English (a language that is written from left to right).

According to the conceptual metaphor theory (Lakoff and Johnson, 1980; Lakoff, 1993), people resort to expressions that refer to concrete concepts when they talk about abstract concepts such as TIME or LOVE and, in doing so, transfer the vocabulary pertaining to a *source domain* to a *target domain*. For instance, people apply the phrasal lexicon related to the concrete concept of journey (source domain) to the intangible concept of love (target domain) (e.g., *We have* ***come a long way*** *since first met*, *We* ***hit a dead-end***, *This relationship is* ***not going anywhere***, *We decided to* ***go our separate ways***). Our language abounds in conceptual metaphors, such as TIME IS MONEY (e.g., *You are* ***wasting*** *my time*, *This will* ***save*** *you hours*, *I have* ***invested*** *a lot of time in our relationship*, *This is not* ***worth*** *my time*), ANGER IS HEAT (e.g., *She is* ***boiling*** *with anger*, *He was* ***fuming***) or ARGUMENT IS WAR (e.g., *Your claims are* ***indefensible***, *He* ***attacked*** *every* ***weak point*** *in my argument*). The fact that conceptual metaphors like these are so ubiquitous is not haphazard: Lakoff and Johnson propose that conceptual metaphors help us structure our thoughts by offering a framework to our conceptualization processes that allows us to map across conceptual domains. One of the most widely used conceptual metaphors is TIME IS SPACE (Clark, 1973; Traugott, 1978; Alverson, 1994; Haspelmath, 1997) (e.g., *The end is* ***getting closer***, *The time for action has* ***arrived***, *The best part is* ***coming up***, *I leave my past* ***behind***, *The deadline is* ***approaching***). Given that time is an intangible and ephemeral concept that cannot be perceived directly by any sensory organ, people from different cultures capture it conceptually using the more concrete source domain of space (Bender and Beller, 2014).

One of the sources for this time-space mapping is our bodily actions. In fact, the abundant literature on *embodied cognition* (Varela et al., 1991; Lakoff and Johnson, 1999; Gibbs, 2003, Gibbs et al., 2004; Gibbs, 2006; Lachaud, 2013) offers substantial support to the idea that many conceptual metaphors arise from (and are grounded in) embodiment. According to the embodied cognition hypothesis, the way our bodies interact with the world and other people affect the way we form and communicate ideas. In other words, our sensorimotor experiences influence the way we think and talk. For instance, the conceptual metaphor MORE IS UP (e.g., *His wage came* ***down***, *The fees went* ***up***) may originate in the many occasions in which our perceptions showed us that greater quantities are usually associated with increased height (e.g., the more papers we pile up, the higher the stack will be). Similarly, the origin of the conceptual metaphor AFFECTION IS WARMTH (e.g., *She gave me a* ***warm*** *greeting*, *She is a* ***cold*** *lady*) may reside in the fact that everybody has experienced physical feelings of warmth during pleasant situations that involved intimacy and comfort (Zhong and Leonardelli, 2008). Indeed, in an experimental study, Williams and Bargh (2008) have demonstrated that physical experiences of warmth unconsciously increase interpersonal feelings of warmth (e.g., a person is more likely to judge the experimenter who gave her a drink as generous and caring if the drink was hot instead of cold; people are more likely to engage in an act of generosity if they were given a hot therapeutic pad instead of a cold one). Likewise, Gibbs (2013) has shown that the conceptual metaphor RELATIONSHIPS ARE JOURNEYS is based on embodied simulations. In his study, participants who read stories about successful romantic relationships that contained metaphors such as *Your relationship was moving along in a good direction* walked longer distances than participants who read stories about unsuccessful relationships (e.g., *You and your wife are stuck in this marriage*); more importantly, this difference in walking distance disappeared when the critical metaphorical statement (e.g., *Your relationship was moving along in a good direction*) was replaced by a non-metaphorical expression (e.g., *Your relationship was very important to you*). Finally, another example of a conceptual metaphor that arises from our sensorimotor experiences is MORALITY IS CLEANLINESS (e.g., *He is involved in some dirty business*). For instance, previous studies have shown that exposure to moral “impurity” increases the desire to get cleansing products and the likelihood of taking antiseptic wipes (Zhong and Liljenquist, 2006). Deceiving acts also increase the likelihood of using hand sanitizer (Lee and Schwarz, 2010). Cleanliness is also associated with more severe judgments on controversial topics such as abortion and pornography (Zhong et al., 2010), and dirty workplaces trigger harsher judgments on other people’s immoral acts (Schnall et al., 2008).

In the case of the conceptual metaphor TIME IS SPACE, which is the focus of the current investigation, the embodied source of this mapping seems to reside in our own kinetic experiences (that is, our bodies or body parts moving) or our perceptions (that is, seeing other entities move). In fact, the concept of time and the concept of space are naturally linked via our movements and vision: When we move our bodies to cover longer distances (or when we observe other people’s bodies do it), we also take more time; moreover, we leave the places we visited first behind, whereas we look ahead while approaching places we will reach soon. This explains why many cultures consider that the past resides in the back and the future in front (e.g., *I always leave my past* ***behind***; *We are* ***moving toward*** *a new digital era*) (although this ego-moving conception of time seems to be impaired in blind people; see Rinaldi et al., 2018), or that upcoming events move toward us until they reach us and move past us, becoming, at that point, past events (e.g., *A new experiment is* ***coming up***) (Wierzbicka, 1973; Bender et al., 2005, 2010, 2012; Bender and Beller, 2014; Rothe-Wulf et al., 2015; Sinha and Bernárdez, 2015; Moore, 2017; Bylund et al., 2020; Callizo-Romero et al., 2020; Feist and Duffy, 2020; Almirabi, 2021). An exception to this “FUTURE IS IN FRONT OF EGO” and “PAST IS IN BACK OF EGO” rule is Aymara language (an Amerindian language spoken in the Andean highlands of western Bolivia, southeastern Peru and northern Chile), whose speakers show linguistic and gestural signs of considering that FUTURE IS BEHIND EGO and PAST IS IN FRONT OF EGO, following the rationale that people can “see” the past, but not yet the future (Núñez and Sweetser, 2006).

Among the many kinetic and perceptual experiences that act as embodied sources of the time-space mapping, our experiences with reading and writing deserve special attention. For instance, Bergen and Lau (2012) demonstrated that English speakers (who write from left to right), mainland China Mandarin speakers (who write from left to right and then top to bottom), and Taiwanese Mandarin speakers (who write from top to bottom and then from right to left) responded differently when they were asked to order sets of cards depicting different stages of development of plants and animals (e.g., tadpole, froglet, frog) in chronological order. English speakers always represented time as moving from left to right. Mainland China Mandarin speakers also had a tendency to use the left to right direction, but there was also a small portion of participants who decided to lay the cards out from top to bottom. In the case of Taiwanese Mandarin speakers, they were just as likely to represent time as moving from left to right as from top to bottom, with a proportion of participants depicting it as moving from right to left. This pattern of results may also be related to the fact that the Chinese temporal words for *last* (e.g., in *last month*) and next (e.g., in *next week*) are, respectively, the words *shàng* (literally meaning *up*) and *xià* (literally meaning *down*) (Scott, 1989; Chen, 2014, 2021; Su, 2019). Other studies have also shown that Mandarin speakers display a vertical top-to-bottom pattern for time, congruent with Chinese vertical spatiotemporal metaphors (Boroditsky, 2001, 2018; Boroditsky et al., 2010). Accordingly, Mandarin-English bilingual speakers are more likely to arrange time vertically the more proficient in Mandarin they are; they are also more likely to do so when tested in Mandarin than when tested in English (Fuhrman et al., 2011). Similarly, Tversky et al. (1991) revealed that the directionality of people’s graphic productions depends on the language they speak: Arabic speakers (who write from right to left) tend to represent temporal concepts from right to left, whereas English speakers tend to do it from left to right. Relatedly, Fuhrman and Boroditsky (2010) revealed that English speakers arranged temporal sequences to progress from left to right, whereas Hebrew speakers (who write from right to left) arranged them from right to left. A similar study (Zhang et al., 2022) revealed that English speakers prefer arrangements that place past-themed photos to the left, whereas that was not the case for Farsi speakers (who write from right to left). Moreover, Fuhrman and Boroditsky (2010) also showed that English speakers had faster reaction times when the key they had to press to indicate that an event happened earlier than another event was located on the left, compared to when the key was located on the right; on the contrary, Hebrew speakers displayed the reverse patterns. Spanish speakers (who write from left to right) also respond more quickly when the past-related words are presented on the left, compared to when they are presented on the right (Santiago et al., 2007). Recall of temporal information is also more accurate when it is presented from left to right than from right to left for English speakers (Fischer-Baum and Benjamin, 2014). Even a study using auditory tasks (Ouellet et al., 2010) has revealed that Hebrew speakers show signs of having temporal representations that differ from Spanish speakers’ even when the stimuli presented does not use the visual modality; in fact, Spanish speakers respond faster to auditorily presented past words with the left hand and to auditorily-presented future words with the right hand, whereas Hebrew speakers show the opposite pattern. Even more interestingly, it has been shown that products’ design and arrangement seem to follow these language-specific patterns of time-space mappings (Pitt and Casasanto, 2022): For instance, in the United States, shampoos tend to be found on the left and conditioners on the right on supermarket shelves, whereas in Israel the opposite arrangement is found.

Despite the abundance of studies addressing different aspects of this time-space mapping, it is striking to note how scarce the literature studying this relationship among Arabic speakers is, despite Arabic being one of the five most spoken languages in the world. In fact, besides the articles mentioned above, very few other studies (mostly theses, dissertations or preprints of qualitative nature) have addressed this matter (Eweida, 2007; Hamdi, 2008; Berrada, 2009; Filipović and Jaszczolt, 2012; Maalej, 2020). What is even more surprising is that there has not yet been a study that investigates how bilingual Arabic speakers (e.g., Arabic-English bilingual speakers) conceptualize this mapping. One of the few previous studies testing bilingual participants is by Miles et al. (2011). These authors revealed that Mandarin-English speakers seem to have both a vertical and a horizontal time-space mapping, consistent with the writing direction in Mandarin and English, respectively. However, in this study, the researchers did not manipulate the language in which participants were tested (i.e., Mandarin or English): All participants were tested in English.

Our study intends to fill this gap by investigating whether bilingual speakers map time onto space differently depending on the language that is activated the most during the experiment. More specifically, we want to compare how Arabic-English bilingual speakers (who use two writing systems with opposing directions) react when asked to map time to space using instructions presented purely in Arabic or purely in English. For this purpose, we asked Arabic-English bilinguals to read a story that was presented either in Arabic or in English, and subsequently arrange a series of cards depicting different scenes of the story in chronological order. This study addresses the larger theoretical goal of whether language activation plays a role on how bilingual speakers map time onto space based on the two writing directions they use.

## Methods

### Participants

A total of 94 Arabic-English bilingual participants were recruited from introductory psychology classes at the American University of Sharjah in exchange for partial course credit. All students in the psychology participant pool had been sent a pre-screening survey that gathered demographic information, and in order to take part of this study, participants had to have rated themselves as “very good” or “excellent” on a 5-point Likert scale that included “1-poor,” “2-fair,” “3-good,” “4-very good,” and “5-excellent” in all eight of the following areas: speaking, oral comprehension, reading and writing in both English and Arabic. 19 participants were removed from all analyses for not following the instructions, leaving 75 participants who were included in the analyses. A total of 39 of these 75 participants were tested in Arabic, and 36 were tested in English.

### Materials

The participants were presented a story either in English (included in Supplementary Appendix A) or in Arabic (included in Supplementary Appendix B). After reading the story out loud, they were asked to arrange nine laminated cards that depicted the different scenes of the story (Quino, 2022; images adapted from https://www.usuariosdelosmedios.es/quino-ser-como-todos/).

### Procedure

Depending on the condition that participants were assigned to, they were fully tested either in English or Arabic. This means that, from the moment participants arrived at the laboratory, they were addressed by fully proficient and balanced English-Arabic bilingual research assistants who greeted them, gave them instructions and debriefed them using exclusively either English or Arabic.

Participants were tested individually. They were instructed to sit at a table and read out loud the story that they were presented. After being given the opportunity to clarify any questions they had regarding the story, they were given a shuffled stack containing nine laminated cards depicting the different scenes of the story that they had just read. Participants were then asked to arrange those nine cards in chronological order. Participants were given the freedom to use all the space on the table they were seated at (which had a width of 95 cm and a length of 55 cm) as they pleased.

**FIGURE 1:**
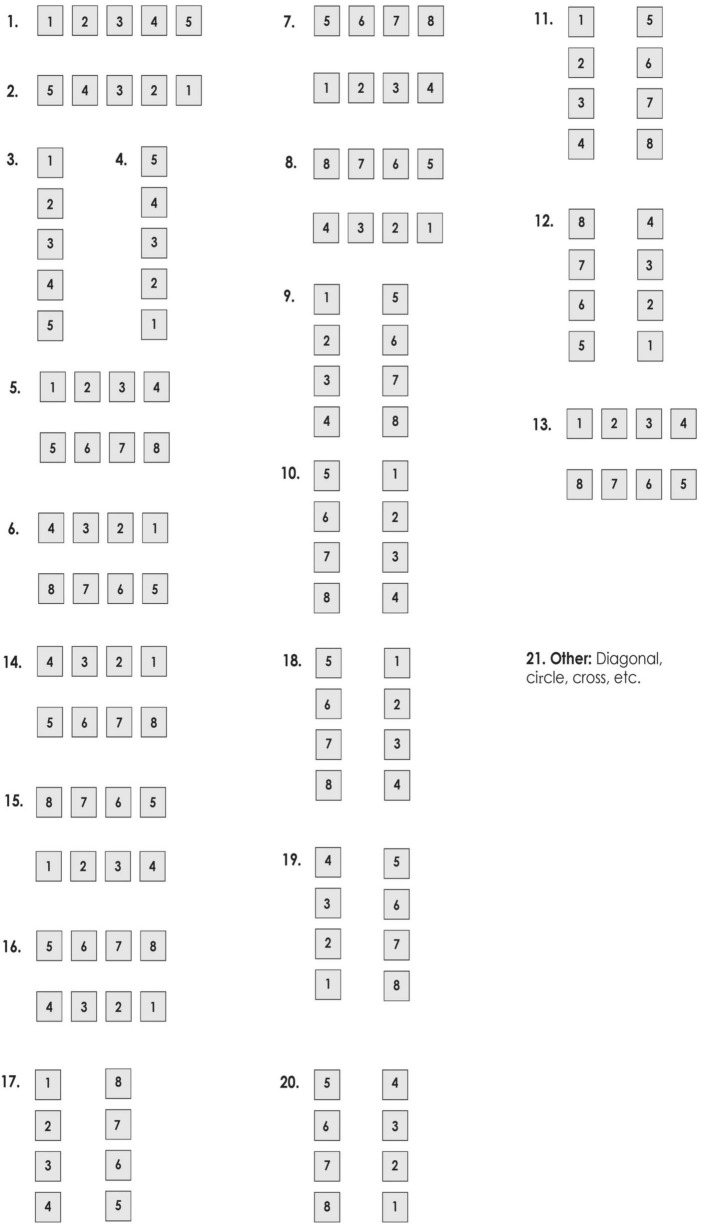
Codes used to classify the way participants arranged the cards.

Each participant’s final card configuration was coded by two independent research assistants who were trained to use the codes presented in Figure 1. In case of inconsistencies between the two coders, either the principal investigator or a senior research assistant intervened to determine the final and most appropriate codes for those cases.

## Results

Figure 1 displays the coding system used for the initial coding. Out of the 39 participants who were tested in Arabic, seven (17.95%) used a purely left to right arrangement (code 1 in Figure 1). Four (10.26%) used a purely right to left arrangement (code 2). Seven (17.95%) used a purely top to bottom arrangement (code 3). A total of 13 (33.33%) used a combination of left to right and top to bottom (code 5). Four (10.26%) used a combination of right to left and top to bottom (code 6). Two (5.13%) used a combination of top to bottom and left to right (code 9). One (2.56%) used a combination of top to bottom and right to left (code 10). One (2.56%) used an L-shaped arrangement that started as purely top to bottom and then switched to purely left to right (code 21).

Out of the 36 participants who were tested in English, eight (22.22%) used a purely left to right arrangement (code 1). Eight (22.22%) used a purely top to bottom arrangement (code 3). A total of 13 (33.33%) used a combination of left to right and top to bottom (code 5). Six (16.67%) used a combination of top to bottom and left to right (code 9). One (2.56%) used an L-shaped arrangement that started as purely top to bottom and then switched to purely left to right (code 21).

The arrangements that were purely vertical were not taken into account in our analysis because they were unrelated to the writing directions in English and Arabic. Thus, the seven participants tested in Arabic and the eight participants tested in English who used the purely top to bottom arrangement (code 3) were not included in our analysis because this arrangement is not relevant for our specific research question (in other words, top-to-bottom arrangements are not reflective of either Arabic or English writing directions). That left us with 32 participants tested in Arabic and 28 participants tested in English who used arrangements that included a horizontal (left to right or right to left) component. These remaining 60 participants’ final arrangements that had a horizontal component were re-coded into two categories: including a left to right component or including a right to left component. That is, participants’ arrangements that were assigned codes 1, 5, 9, and 21 were re-coded into the category “includes left to right component,” and participants’ arrangements that were assigned codes 2, 6, and 10 were re-coded into the category “includes right to left component.”

Out of these 32 participants tested in Arabic that were included in our analysis, 23 (71.88%) used arrangements that included a left to right component, whereas nine (28.13%) used arrangements that included a right to left component. On the other hand, all of the 28 participants tested in English that were included in our analysis used arrangements that included a left to right component.

The Fisher’s exact test, conducted using the function *tabi* in Stata 18, indicated that participants tested in Arabic were significantly more likely to use arrangements that included a right to left component, compared to participants tested in English (*p* = 0.001).

## Discussion

The results show that when Arabic-English bilingual speakers are in “Arabic mode,” they are more likely to use chronological arrangements that include the right-to-left directionality (in agreement with the Arabic writing direction), compared to when they are in “English mode.” Arrangements that included the right-to-left directionality were only used by participants tested in Arabic, and never by participants tested in English. This suggests that when Arabic-English bilingual speakers have the Arabic language more activated than the English language (due to, for instance, having an interlocutor who talks to them solely in Arabic, or having recently been exposed to text in Arabic), they are more likely to conceptualize time as moving from right to left (following the Arabic writing direction), compared to when they have the English language more activated.

Three main conclusions can be drawn from these results. First, this study contributes to the scarce body of literature that suggests that the embodiment of time does not depend only on innate basic sensorimotor abilities, such as vision and walking (as in the case of the time-space mapping that equates the past to the back and the future to the front, derived from our visual and motor experiences of moving in space and leaving locations visited in the past behind), but also on more complex and uniquely human sensorimotor abilities, such as writing, which, in human evolution, is a relatively recent skill that has been around for only around 5,000 years.

Second, this study is the first that has demonstrated that the embodiment of the time-space mapping among bilingual speakers is not fixed but is rather context-dependent: Language activation influences how bilingual speakers map time onto space, and how they reflect this mapping through hand movements, such as the ones used to arrange images depicting different scenes of a story in chronological order. In fact, to our knowledge, this is the first study that has tested how time is mapped onto space in bilingual speakers by manipulating language activation. These results expand on Hendricks and Boroditsky’s (2017), who revealed that it is possible to teach new time-space metaphors (e.g., vertical linguistic metaphors, such as *Breakfast is above dinner* or *Breakfast is below dinner*) to English speakers, who subsequently displayed new non-linguistic space-time mappings through implicit association tasks.

Third, the finding that even though the participants tested in Arabic were significantly more likely to equate the past to the right and the future to the left compared to the participants tested in English, the majority of the participants tested in Arabic still used arrangements that included the left-to-right directionality (in agreement with the English writing direction) suggests that the linguistic characteristics of the environment (such as daily exposure to a certain language) also influences the application of time-space metaphors. In particular, the tendency to use left-to-right directionality might be related to the characteristics of our sample, which was composed of fully bilingual Arabic-English speakers who had been highly exposed to the American culture (e.g., American series, music) and the American style of education since childhood. In fact, given that this study was conducted at the American University of Sharjah, whose language of instruction is English, it is logical to expect weaker effects of Arabic language activation during the experiment. This aspect of the results suggests that it would be fruitful for future studies to replicate this experiment using a sample of equally fluent Arabic-English bilingual speakers who live in a more Arabic-dominant place to examine the role of linguistic environment more in detail. Finally, we must also take into account that not all written Arabic follows the right-to-left direction: In fact, Arabic numbers are written from left to right. Therefore, the way Arabic-English bilinguals represent the progression of mathematical operations in space in both Arabic and English may have also influenced the way in which they think time moves (for more information about the representation of mathematical concepts in bilingual speakers see Farsani, 2016).

In conclusion, we found that writing direction and language activation influence the use of conceptual metaphors. In particular, we demonstrated that the language in which bilingual Arabic-English speakers were tested influenced how they mapped time onto space when recalling the order of events in a story. Our results are consistent with theories that suggest that the embodied source of the TIME IS SPACE metaphor resides in kinetic experiences. Importantly, all our participants lived and worked in the same environment and, thus, the observed differences can be attributed to the language that was activated specifically during testing. However, even so, we also observed an overall bias toward using a spatial arrangement that was consistent with the language that was most often used in the university environment (English). In conclusion, the application of conceptual metaphors appears to be a product of both immediate linguistic context (i.e., the language used during the testing session) and general linguistic context (i.e., the language that is the most dominant in the environment participants live in).

## Data availability statement

The raw data supporting the conclusions of this article will be made available by the authors, without undue reservation.

## Ethics statement

The studies involving humans were approved by the IRB, American University of Sharjah. The studies were conducted in accordance with the local legislation and institutional requirements. The participants provided their written informed consent to participate in this study.

## Author contributions

JP: Conceptualization, Data curation, Formal Analysis, Investigation, Methodology, Project administration, Resources, Supervision, Visualization, Writing – original draft, Writing – review & editing. CG: Data curation, Formal Analysis, Supervision, Writing – original draft, Writing – review & editing. TS: Data curation, Formal Analysis, Supervision, Writing – original draft, Writing – review & editing.
