# Supplementary material for: Writing direction and language activation affect how Arabic-English bilingual speakers map time onto space
Source: Front Psychol. 2024 Jan 24;14:1356039. doi: 10.3389/fpsyg.2023.1356039 (PMC10849100; doi:10.3389/fpsyg.2023.1356039)
Supplement: Supplementary file 1 [file Data_Sheet_1.docx]

Appendix A

*Story Presented to Participants Tested in English*

**Kids these days: What is normal?**

One day, Ms. Smith saw her 9-year-old daughter, Lisa, calmly playing with her dolls.

The next day, she saw Lisa sewing a dress for her dolls.

Later, she saw Lisa wisely reading some science books.

Lisa’s behavior got Ms. Smith concerned: How come Lisa didn’t watch TV at all? Was she normal? Why wasn’t Lisa like all the other kids, who begged for more TV time? Ms. Smith was so worried that she decided to take Lisa to the doctor.

The doctor checked Lisa’s legs. Everything seemed fine.

Then, the doctor checked Lisa’s arms. Everything seemed fine there too.

The doctor wondered why Lisa was not like all the other kids. He started to be concerned too. He took some time to reflect on the best possible solution for Lisa’s “problem”.

Suddenly, the doctor had an amazing idea: Using his medical hammer, he hit Lisa on her head really hard!

The next morning, Ms. Smith found Lisa in the living room watching TV, drinking soda and chewing gum, just like any other “normal” kid her age. Finally, Ms. Smith felt relieved.

Appendix B

*Story Presented to Participants Tested in Arabic*

في يوم من الأيام، رأت السيدة سميث إبنتها ليزا الذي عمرها تسعة سنوات، تلعب بهدوء مع ألعابها.

في اليوم التالي ، رأت ليزا تخيط فستانًا لدميتها.

في وقت لاحق، رأت ليزا وهي تقرأ بعض الكتب العلمية بحكمة.

تصرف  ليزا اهمم السيدة سميث: لماذا ليزا لا تشاهد التلفاز أبدا؟ هل هي طبيعية؟ لماذا  ليزا ليست كالأطفال الآخرين الذين يترجون لوقت أكثر لمشاهدة التلفاز؟ السيدة سميث قلقت جدا و قررت أن تأخذ  ليزا للطبيب.

الطبيب فحص أرجل  ليزا. كل شيء كان على ما يرام.

ثم، الطبيب فحص أذرع  ليزا. كل شيء كان على ما يرام أيضا.

تساءل الطبيب لماذا لم تكن ليزا مثل جميع الأطفال الآخرين. بدأ يشعر بالقلق أيضا. لقد استغرق بعض الوقت للتفكير في أفضل حل ممكن لـ "مشكلة" ليزا.

فجأة ، خطرت للطبيب فكرة رائعة: باستخدام مطرقة طبية ، ضرب ليزا على رأسها بشدة!

في صباح اليوم التالي، وجدت السيدة سميث ليزا في غرفة المعيشة تشاهد التلفاز وتشرب الصودا تمضغ العلكة ، تمامًا مثل أي طفل "طبيعي" آخر في عمرها. أخيرًا ، شعرت السيدة سميث بالارتياح.
